# Ultrasound-guided targeted biopsies of CT-based radiomic tumour habitats: technical development and initial experience in metastatic ovarian cancer

**DOI:** 10.1007/s00330-020-07560-8

**Published:** 2020-12-14

**Authors:** Lucian Beer, Paula Martin-Gonzalez, Maria Delgado-Ortet, Marika Reinius, Leonardo Rundo, Ramona Woitek, Stephan Ursprung, Lorena Escudero, Hilal Sahin, Ionut-Gabriel Funingana, Joo-Ern Ang, Mercedes Jimenez-Linan, Tristan Lawton, Gaurav Phadke, Sally Davey, Nghia Q. Nguyen, Florian Markowetz, James D. Brenton, Mireia Crispin-Ortuzar, Helen Addley, Evis Sala

**Affiliations:** 1 Department of Radiology, University of Cambridge, Cambridge, CB2 0QQ UK; 2 Department of Biomedical Imaging and Image-guided Therapy, Medical University Vienna, 1090 Vienna, Austria; 3 Cancer Research UK Cambridge Centre, University of Cambridge, Cambridge, CB2 0RE UK; 4 Cancer Research UK Cambridge Institute, University of Cambridge, Cambridge, CB2 0RE UK; 5 Cambridge University Hospitals NHS Foundation Trust, Addenbrooke’s Hospital, Cambridge, CB2 0QQ UK; 6 Department of Pathology, Addenbrooke’s Hospital, Cambridge, UK; 7 Canon Medical Research Europe Ltd, Edinburgh, UK; 8 Canon Medical Systems LTD, Crawley, UK; 9 Information Engineering Division, Department of Engineering, University of Cambridge, Cambridge, CB2 1PZ UK

**Keywords:** Ovarian neoplasms, Radiomics, Computed tomography

## Abstract

**Purpose:**

To develop a precision tissue sampling technique that uses computed tomography (CT)–based radiomic tumour habitats for ultrasound (US)-guided targeted biopsies that can be integrated in the clinical workflow of patients with high-grade serous ovarian cancer (HGSOC).

**Methods:**

Six patients with suspected HGSOC scheduled for US-guided biopsy before starting neoadjuvant chemotherapy were included in this prospective study from September 2019 to February 2020. The tumour segmentation was performed manually on the pre-biopsy contrast-enhanced CT scan. Spatial radiomic maps were used to identify tumour areas with similar or distinct radiomic patterns, and tumour habitats were identified using the Gaussian mixture modelling. CT images with superimposed habitat maps were co-registered with US images by means of a landmark-based rigid registration method for US-guided targeted biopsies. The dice similarity coefficient (DSC) was used to assess the tumour-specific CT/US fusion accuracy.

**Results:**

We successfully co-registered CT-based radiomic tumour habitats with US images in all patients. The median time between CT scan and biopsy was 21 days (range 7–30 days). The median DSC for tumour-specific CT/US fusion accuracy was 0.53 (range 0.79 to 0.37). The CT/US fusion accuracy was high for the larger pelvic tumours (DSC: 0.76–0.79) while it was lower for the smaller omental metastases (DSC: 0.37–0.53).

**Conclusion:**

We developed a precision tissue sampling technique that uses radiomic habitats to guide in vivo biopsies using CT/US fusion and that can be seamlessly integrated in the clinical routine for patients with HGSOC.

**Key Points:**

*• We developed a prevision tissue sampling technique that co-registers CT-based radiomics–based tumour habitats with US images.*

*• The CT/US fusion accuracy was high for the larger pelvic tumours (DSC: 0.76–0.79) while it was lower for the smaller omental metastases (DSC: 0.37–0.53).*

## Introduction

Improving patient stratification is a major challenge in high-grade serous ovarian cancer (HGSOC) where both genomic and tumour microenvironment heterogeneity is found within and between patients [1–4]. High genomic heterogeneity is associated with reduced progression-free survival [1, 2, 4–7].

Molecular pathology has become key in improving stratification, but single biopsies fail to assess spatial tumour heterogeneity, providing inadequate sampling of the multiscale complexity of the disease. However, since the number of biopsies that can be obtained from a tumour is limited due to the invasiveness of the procedure, there is a need to guide this sampling. Routinely performed medical scans provide a non-invasive solution for capturing spatial heterogeneity quantitatively by the use of radiomics [8], even offering the possibility of doing so in a longitudinal manner if acquired over the course of therapy.

Radiomics refers to the analysis of quantitative features extracted from imaging data [8–10]. The analysis of radiomic features in a spatial manner is often performed by extracting tumour habitats. Tumour habitats are defined as regions with distinct local radiomic phenotypes (i.e. texture features expression), which may capture different pathophysiology [11, 12]. Tumour habitats can be identified on variable imaging modalities including computed tomography (CT), magnetic resonance imaging (MRI), or ultrasound (US). These tumour habitats may represent areas of different genomic and transcriptomic characteristics [12] and could be used to understand tumour resistance to targeted therapeutics. Indeed, some associations have already been found between spatial radiomics and biological correlates [13, 14]. We have developed patient-specific 3D-printed custom moulds to enable precise multiregional sampling of different radiomic regions from resected specimens [12, 15] as targeted biopsies are key to capture relevant tumour regions. However, methods to sequentially sample specific radiomic habitats during therapy have not been developed.

MRI or CT/US fusion biopsies are an emerging technique to selectively target areas of interest [16, 17]. MRI/US fusion biopsies improve the accuracy to detect especially clinically significant prostate cancer while decreasing the detection of low-grade cancers [16, 18]. MRI or CT/US fusion systems are increasingly used to target hepatic lesions as they increase the accuracy to target tumours that are undetectable with US alone [19]. So far no studies have applied imaging-guided US fusion biopsies in patients with HGSOC.

The purpose of this study was to develop a precision tissue sampling technique that uses CT-based radiomic tumour habitats for US-guided targeted biopsies that can be integrated in the clinical workflow of patients with HGSOC.

## Material and methods

**Fig. 1:**
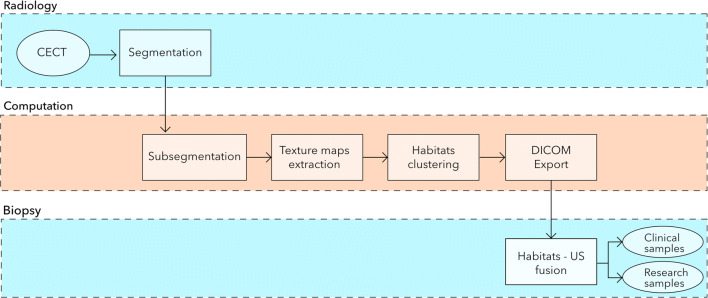
Schematic workflow of the proposed US-guided targeted biopsies of distinct CT-based radiomic tumour phenotypes. Standard-of-care contrast-enhanced (CE) CTs were used to manually segment US targetable tumour deposits in the pelvis or omentum. Automated tissue sub-segmentation was applied in selected omental lesions to remove non-tumoural fatty tissue at the segmentation edges. Spatial radiomic features were computed and the Gaussian mixture modelling clustering was applied to identify up to three habitats per tumour lesion. Habitat maps were exported and manually uploaded together with the source CT data to a US machine. Up to two biopsies per habitat were obtained and used for clinical diagnosis, as well as for research purposes

This single-centre prospective study was approved by our institutional review board. Written and informed consent was obtained from all participants. The study flowchart is shown in Fig. 1.

### Study participants

Research participants were consecutively and prospectively recruited from the Cambridge University Hospital, between September 2019 and March 2020. Inclusion criteria were age of 18 years or higher; radiological, clinical, or biochemical suspicion of HGSOC; ability to undergo US-guided biopsy of an omental or pelvic lesion; contrast-enhanced CT of the abdomen and pelvis; and willingness and ability to participate. Exclusion criteria included inability to undergo US-guided biopsy (platelet count < 50 G/l; prothrombin time > 16 s), ongoing treatment with anticoagulation (warfarin or rivaroxaban), and targetable tumour volume of less than 3 cm^3^. Eight patients met the inclusion criteria. In two patients, we were not able to perform the US-guided fusion biopsy as the dedicated US machine was not available at the biopsy appointment.

### CT acquisition and tumour segmentation

The clinically indicated contrast-enhanced CT scans were acquired on three different scanners with slice thickness ranging between 2 and 5 mm (Table 1). Images of the portal venous phase, reconstructed with the soft tissue reconstruction, were used for tumour segmentation. After the identification of a potentially targetable lesion, it was manually segmented to create a volume of interest (VOI) using the Microsoft Radiomics App V1.0.28434.1 (project InnerEye https://www.microsoft.com/en-us/research/project/medical-image-analysis, Microsoft) by a radiologist in the 5th year of training (L.B.) under the supervision of a board-certificated radiologist with special expertise in ovarian cancer imaging (E.S.) with 17 years of experience.

**Table 1:** CT imaging parameter settings

ID	Vendor	CT machine	Row	Tube voltage (kV)	Pixel spacing (mm)	Slice thickness (mm)	Kernel	Reconstruction interval (mm)	Delay (sec)	Contrast type	Contrast (ml)	Contrast dose (ml/kg)
1	GE	Optima CT660	64	100	0.814	3.75	Standard	1.5	60	Omnipaque 300	60	1.0
2	GE	Optima CT660	64	120	0.672	3.75	Standard	2.5	60	Omnipaque 300	70	1.0
3	Siemens	Definition AS	64	140	0.977	2	I30f\3	1.5	60	Omnipaque 300	70	0.9
4	Siemens	Definition AS	64	100	0.596	2	I26f\3	1.5	60	Omnipaque 300	70	1.0
5	Siemens	Definition Flash	128	100	0.727	2	B20f	1.5	60	Omnipaque 300	60	0.9
6	Siemens	Definition Flash	128	120	0.719	5	B30f	5	60	Omnipaque 300	70	0.7

*CT*, computed tomography

### Computational work to obtain CT radiomic habitats

In two patients with omental disease (patients 3 and 4), we performed an automatic sub-segmentation (i.e. delineation of the solid region of the tumour excluding other parts such as fat) of the omental VOI as proposed recently in [20]. The sub-segmentation was only used for two omental tumours as pelvic lesions do not contain interspersed fat and are better defined.

Radiomic feature maps were created for each of the VOIs. Radiomic maps differ from traditional radiomic features in that textures are computed for the neighbourhood around each and every voxel in the VOI; the resulting “maps” therefore capture the spatial variation of the texture lesion. In particular, we used a sliding window algorithm with a window size of 5 × 5 voxels. Textures extracted included grey-level co-occurrence matrix energy, entropy, sum average, correlation, inverse difference moment normalised, contrast, cluster shade, cluster prominence, and Haralick correlation [21]. Patch-wise texture maps are done by calculating the Haralick texture values in sliding windows centred around each voxel. The sliding window used was 5 × 5 voxels in our case. Texture values were extracted using 32 grey levels and 2D directionality using the Computational Environment for Radiological Research (CERR) package (10.1118/1.1568978). To avoid redundancy arising from the mathematical formulation of radiomic features, we used the principal component analysis (PCA), a technique for dimensionality reduction. The texture maps were mapped into six principal components (PC) using PC analysis that retained more than 90% of the variance in an independent cohort of 75 patients with HGSOC undergoing pre-neoadjuvant therapy. The principal component version of the texture features maps and Hounsfield unit values were used to define habitats using a clustering technique known as the Gaussian mixture modelling. The maximum possible number of habitats was set to 3, in agreement with the maximum number of targeted biopsies per lesion considered to be feasible. The optimal number of habitats was automatically selected according to the minimum Akaike information criterion (AIC) value. The AIC is a measure of model quality that can be used for comparing clustering results. The lower AIC represents the maximum accuracy achieved. Habitat maps resulting from the clustering step were then exported in DICOM format. Computations were performed with MATLAB^®^ R2019b (The MathWorks).

### US-guided biopsy

All participants underwent a US-guided biopsy using the Aplio i800 US system (Canon Medical Systems, Otawara, Japan) with an i8CX1 3.5-MHz convex transducer (PVI-475BX; Canon Medical Systems) by a board-certificated gynaecological radiologist (H.A.) with 9 years of experience. The US machine was coupled with a magnetic field generator and an electromagnetic position sensor connected with a position-sensing unit attached to the US probe through a bracket. The commercially available software Smart Fusion (Canon Medical Systems) was used to achieve real-time image fusion of the US with the CT data.

Before starting the US biopsy procedure, the CT images were registered onto the US data using a landmark-based rigid-body registration. First, the axial orientation of the CT images was registered by obtaining a US image in a strictly axial plane. Second, between one and three fusion points were used as landmarks to register the *z*-axis between the CT volume dataset and the US data. The first fusion point in all patients was the anterior superior margin of the pubic symphysis. The umbilicus and spina iliaca anterior superior were used as second and third fusion points. The fusion quality indicator was between 8 and 10. This can take a value between 1 and 10, where 1 indicates a poor fusion signal quality and 10 an excellent quality.

Up to six biopsies (two per tumour habitat) were obtained using a 14G biopsy needle (Temno Evolution Biopsy Device, Cardinal Health). Cine clips covering the targeted tumour tissue were recorded before and during the biopsy procedures.

Half of each biopsy core was formalin embedded. After the biopsy, we monitored patients for 6 h before discharge. Adverse events including bleeding, wound infection, and re-hospitalisation were assessed.

### Quantification of CT/US fusion accuracy

The dice similarity coefficient (DSC) was used to quantify the fusion accuracy by assessing the overlap of the tumour region between the US and CT. The co-registered CT/US image covering the largest tumour area on the CT was selected to calculate the DSC. These images were exported as JPEG files. A radiologist in the 5th year of training (L.B.) segmented the tumour on the B-mode US image using ImageJ (ImageJ 1.52a). The binary masks of the segmentations were further processed with MATLAB^®^ R2019b and the DSC was calculated.

### Histological examination

All tissue samples were assessed by a board-certified gynaecologic pathologist (M.J-L).

## Results

### CT/US fusion for radiomic habitat-guided biopsy

**Fig. 2:**
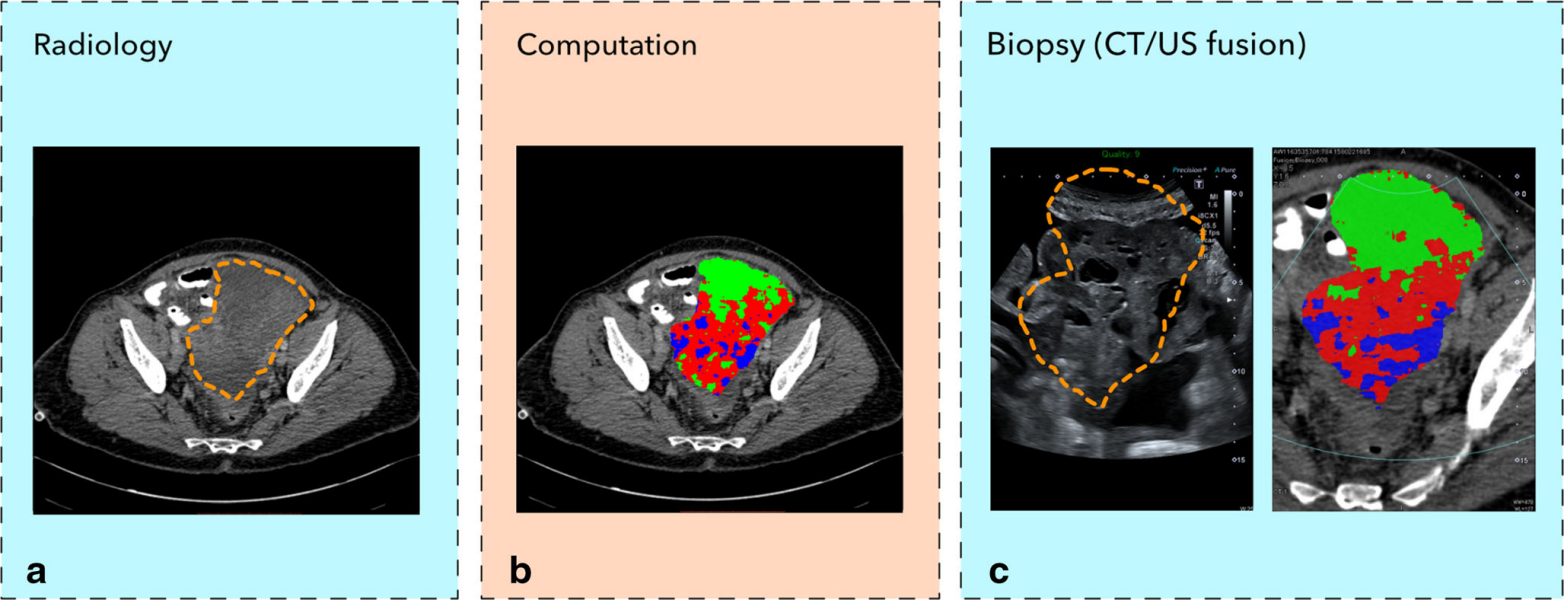
Illustration of a patient with a pelvic tumour. **a** Routine contrast-enhanced CT images were used to manually segment the pelvic tumour (dashed line). **b** Spatial radiomic feature extraction and generation of habitat maps. For this patient, three tumour habitats are feasible and highlighted in blue, red, and green, respectively. **c** The left figure shows the US image with the co-registered CT-based tumour segmentation (dashed line). The right figure shows the CT scan overlaying the US plane, with the habitat maps highlighted in colour. The US images correspond to a different plane orientation with respect to panels **a** and **b**

Figure 1 summarises the radiological and clinical workflow. We performed targeted CT/US fusion-guided biopsies in six patients. The demographic and clinical characteristics are shown in Table 2. Figure 2 displays the detailed imaging characteristics at each step for patient 5. For patients 2 and 5, we obtained biopsies from the pelvic lesion, and in patients 1, 3, 4, and 6, we obtained biopsies from the omental deposits. The tumour volumes of the targeted omental lesions (median = 103 cm^3^; range: 16–295 cm^3^) were smaller compared to those of the pelvic lesions (median = 520 cm^3^; range: 448–592 cm^3^). No adverse events were observed following the biopsy procedures. For patients 1 and 3, the biopsy material was insufficient for diagnosis and these patients underwent diagnostic laparoscopy that established the diagnosis of HGSOC.

**Table 2:** Patient demographics and clinical parameters

ID	Age	Stage	Days Bx-CT	Site of Bx	Nr of biopsies	Nr of clusters	Volume cm^3^	Biopsy result	Weight (kg)
1	59	3C	30	Omentum	1	3	15.7	Insufficient material	61
2	59	4B	10	Pelvis	3	3	591.7	HGSOC	69
3	53	4A	29	Omentum	2	3	295.2	Insufficient material	77
4	68	3C	16	Omentum	4	3	19.4	HGSOC	72
5	76	4A	7	Pelvis	4	3	448.3	HGSOC	66
6	77	4B	26	Omentum	3	3	187.4	HGSOC	94

*Bx*, biopsy; *CT*, computed tomography; *HGSOC*, high-grade serous ovarian cancer

### Assessment of the US-CT fusion accuracy

**Fig. 3:**
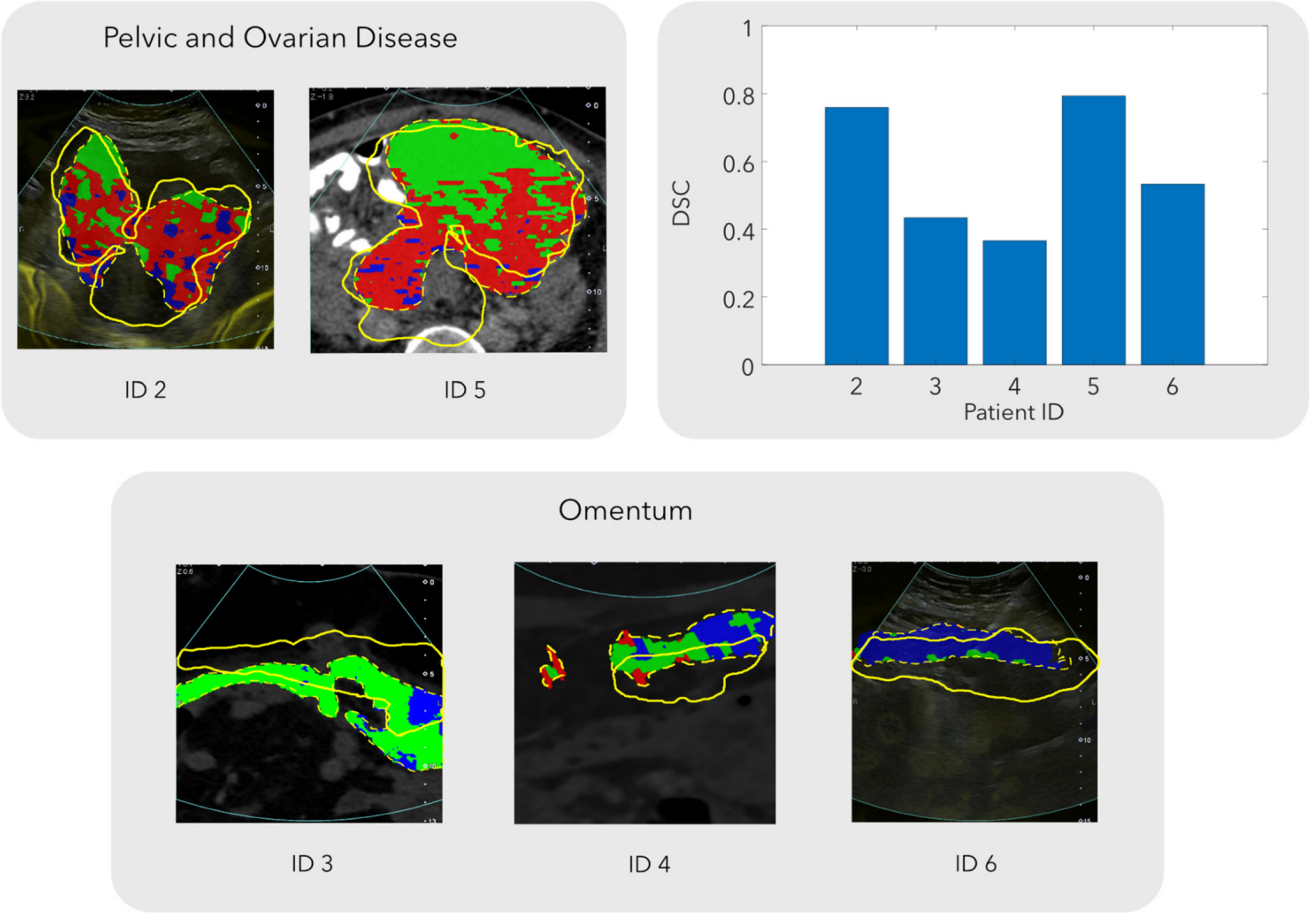
The accuracy of the CT/US fusion was associated with the tumour volume and was higher for pelvic compared to omental tumours. The dice similarity coefficient (DSC) was used to assess the accuracy of the CT/US fusion. The DSC was higher for the pelvic (larger) compared to that for the omental (smaller) tumours that yielded a low DSC. Dashed line, CT segmentation; solid line, US segmentation

The DSC was 0.76 and 0.79, respectively, for pelvic lesions, and 0.37, 0.43, and 0.53 respectively for omental lesions (Fig. 3). We were not able to calculate a DSC for patient 1 as the tumour edges were not visible on the B-mode ultrasound images.

### Histological assessment

**Fig. 4:**
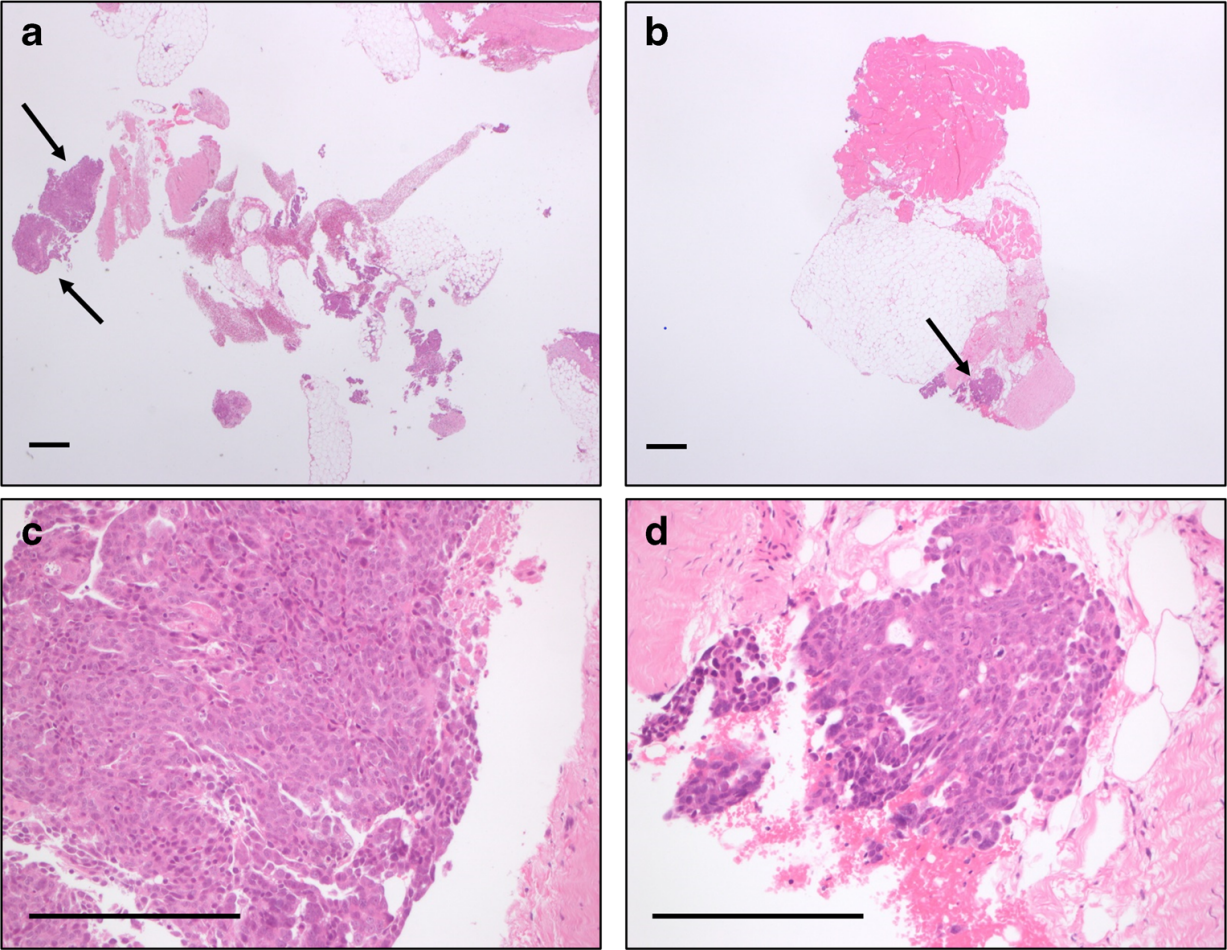
Histological analysis of biopsy material from patient 5 from two habitats. H&E staining of samples from two distinct habitats from the ovarian tumour deposit (arrow) in the same patient. Panels **a** and **b** show low power views of the needle cores. Arrows indicate high power view in **c** and **d**. Bar = 50 μm

In patient 5, we obtained sufficient tumour tissue from two radiomically different habitats to evaluate their morphology using H&E staining (Fig. 4). No differences in terms of tissue morphology were observed between the two tumour habitats.

## Discussion

In this technical development study, we demonstrated the feasibility of prospective sampling of CT-based radiomic habitats using US-guided fusion biopsies in patients with HGSOC prior to neoadjuvant chemotherapy. We used standard-of-care, contrast-enhanced CT to extract radiomic tumour habitat maps and identify distinct regions within the tumour. We proposed a technique to register these CT radiomic tumour habitat maps to the real-time US scans and used them to guide tissue sampling of the habitats.

Radiomic features are a non-invasive method to quantify and map tumour heterogeneity. They are associated with genomic heterogeneity [12, 22, 23] which predicts response to chemotherapy and poor outcome in patients with HGSOC [1, 6]. However, the biological pathways underlying different imaging habitats are poorly understood. To overcome this challenge and lay the foundation for future risk stratification, we proposed a technology that uses standard-of-care CT imaging to sample regions with distinct radiomic habitats.

The accurate spatial registration between the CT and US images is a prerequisite for reliable habitat-guided tissue targeting and for the generalizability of the results. We observed differences in the fusion accuracy between omental deposits and pelvic lesions, with higher accuracies for the pelvic lesions. The average DSC for the pelvic lesions was 0.78, indicating a good registration accuracy. This can be explained by the larger tumour volume of pelvic lesions and by their relatively fixed position in the pelvis compared to more flexible positions of omental deposits. The lower performance in all three omental lesions was due to a high degree of misregistration in the anterior-posterior axis. This is mainly explained by the variability of the anterior abdominal wall and omentum due to the local pressure of the ultrasound probe. Patient tracker systems that correct for local patient movements can overcome this limitation and improve the registration accuracy for difficult tumour sites such as omentum. We plan to implement and test these systems in subsequent studies.

Obtaining high-quality tissue samples that are sufficient for routine workup and genomic analysis are critical to translate research biopsy techniques into clinical practice. The reported success rate for omental and pelvic mass biopsies in patients with suspected ovarian cancer is approximately 89% [24]. Biopsies in two out of six patients (33%) reported in this study yielded an inadequate sample (fat or skeletal muscle) which is higher compared to previous reports. These two patients with inadequate biopsy samples had either a small omental tumour volume (patient 1: 15.7 cm^3^) or a high body mass index that made the US biopsy procedure more challenging.

Our approach has several limitations that can be improved in subsequent studies. We used rigid co-registration and there was a time delay between the CT acquisition and the US-guided biopsy, which means that there could be biases due to unexpected deformations. In addition, the accuracy assessment was based on a limited number of 2D slices, which may not optimally represent the tumour volume. We also found that we were not able to biopsy all the CT-based tumour habitats, because of their small volume. This trade-off between computational precision and practical feasibility and safety will need to have a clinical decision. Based on this work, we recommend setting a threshold of 3 cm^3^ to determine tumour habitats in patients with HGSOC and to generate no more than three habitats per targetable lesion. In addition, we used three different CT scanners with slice thicknesses ranging from 2 to 5 mm and four different reconstruction kernels which could influence radiomics. However, as the aim was to develop a method to co-register radiomic habitat maps to US images to guide tissue sampling, we believe that differences in slice thickness do negatively affect the results of this study. The data we have obtained so far demonstrates the feasibility of the technique but is limited in assessing the molecular differences between tumour habitats. These radiogenomic associations can now be tested in larger studies.

In conclusion, we developed a tissue sampling technique to target CT-based radiomic habitats in vivo using a CT/US fusion technology. This will enable new approaches to discover and validate radiogenomic biomarkers.

## Abbreviations

CT: Computed tomography
DSC: Dice similarity coefficient
HGSOC: High-grade serous ovarian cancer
US: Ultrasound
VOI: Volume of interest
